# Longitudinal change of serum exosomal miR-186-5p estimates major adverse cardiac events in acute myocardial infarction patients receiving percutaneous coronary intervention

**DOI:** 10.3389/fcvm.2024.1341918

**Published:** 2024-04-17

**Authors:** Lingyun Ren, Wei Liu, Shanshan Chen, Haibo Zeng

**Affiliations:** ^1^ Anesthesiology Department, The Central Hospital of Wuhan, Tongji Medical College, Huazhong University of Science and Technology, Wuhan, China; ^2^ Key Laboratory for Molecular Diagnosis of Hubei Province, The Central Hospital of Wuhan, Tongji Medical College, Huazhong University of Science and Technology, Wuhan, China

**Keywords:** acute myocardial infarction, exosomes, microRNA-186-5p, percutaneous coronary intervention, major adverse cardiac events

## Abstract

**Objective:**

Our recently published study discovers that exosomal microRNA (miR)-186-5p promotes vascular smooth muscle cell viability and invasion to facilitate atherosclerosis. This research aimed to explore the prognostic implication of serum exosomal miR-186-5p in acute myocardial infarction (AMI) patients receiving percutaneous coronary intervention (PCI).

**Methods:**

One hundred and fifty AMI patients receiving PCI and 50 healthy controls (HCs) were screened. Serum exosomal miR-186-5p was detected by reverse transcriptase-quantitative polymerase chain reaction assay in AMI patients at admission and after PCI, as well as in HCs after enrollment. Major adverse cardiac events (MACE) were recorded during follow-up in AMI patients receiving PCI.

**Results:**

Serum exosomal miR-186-5p was raised in AMI patients vs. HCs (*P *< 0.001). Besides, serum exosomal miR-186-5p was positively linked to body mass index (*P *= 0.048), serum creatinine (*P *= 0.021), total cholesterol (*P *= 0.029), and C-reactive protein (*P *= 0.018); while it was reversely linked with estimated glomerular filtration rate (*P *= 0.023) in AMI patients. Interestingly, serum exosomal miR-186-5p was correlated with the diagnosis of ST-segment elevation myocardial infarction (*P *= 0.034). Notably, serum exosomal miR-186-5p was decreased after PCI vs. at admission (*P *< 0.001). The 6-, 12-, 18-, and 24-month accumulating MACE rates were 4.5%, 8.9%, 14.8%, and 14.8% in AMI patients. Furthermore, serum exosomal miR-186-5p ≥3.39 (maximum value in HCs) after PCI (*P *= 0.021) and its decrement percentage <median (35%) decrement (*P *= 0.044) estimated elevated MACE in AMI patients.

**Conclusion:**

Serum exosomal miR-186-5p is reduced after PCI, and its post-PCI high level or minor decrease estimates increased MACE risk in AMI patients.

## 1 Introduction

Acute myocardial infarction (AMI) is the most fatal type of coronary artery disease, which is sorted into ST-segment elevation myocardial infarction (STEMI) and non-STEMI (NSTEMI) (1). Percutaneous coronary intervention (PCI) is a fundamental therapy for AMI patients, which has enhanced the clinical outcomes of these patients (2). Unfortunately, major adverse cardiac events (MACE), such as cardiac death, cardiac arrest, recurrent myocardial infarction, and repeat revascularization, occur even after successful PCI, which severely affects the clinical outcomes of AMI patients (3, 4). It is estimated that the incidence of 2-year MACE after PCI is approximately 16%, and the incidence of 3-year MACE after PCI increases to nearly 20% in AMI patients (5–8). Therefore, seeking potential markers that predict MACE may be vital to enhance the management of AMI patients receiving PCI.

Exosomes are enclosed by a lipid membrane bilayer, which is essential in mediating cell-to-cell communication, and microRNA (miR) is the most numerous cargo molecule in the exosomes (9, 10). Of note, exosomal miR, such as miR-21-3p, miR-182-5p, and miR-27b-3p, plays a fundamental role in atherogenesis, which is responsible for the pathology and progression of cardiovascular diseases (11–14). Regarding miR-186-5p, a previous study indicates that serum exosomal miR-186-5p shows a good diagnostic performance for AMI, and its dysregulation in exosomes contributes to atherosclerosis by targeting lectin-like ox-LDL receptor-1 (LOX-1) (15). Additionally, another study reports that serum exosomal miR-186-5p is positively associated with lipid level, coronary stenosis degree, and MACE risk in coronary heart disease patients (16). Inspiringly, our recently published study observes that macrophages-delivered exosomal miR-186-5p inactivates the Src homology 2-containing inositol 5-phosphatase 2 (SHIP2)-mediated phosphatidylinositol 3-kinase (PI3 K)/protein kinase B (AKT)/mammalian target of rapamycin (mTOR) pathway to facilitate vascular smooth muscle cell (VSMC) viability and invasion, thereby promoting atherogenesis (17). Given the above evidence, we hypothesize that exosomal miR-186-5p would possess prognostic implications in AMI patients receiving PCI. However, relevant evidence is scarce.

Accordingly, the current research intended to investigate the relationship of serum exosomal miR-186-5p with MACE in AMI patients receiving PCI.

## 2 Methods

### 2.1 Study population

A total of 150 AMI patients receiving PCI treatment between January 2021 and May 2023 were continuously included in this study. The inclusion criteria were: (i) diagnosed as AMI per guideline from the American Heart Association/American College of Cardiology Joint Committee (18); (ii) ≥18 years old; (iii) treated with PCI; (iv) willing to cooperate with blood sample collection. The exclusion criteria were: (i) with malignant cancers or hematological malignancies; (ii) with severe liver or kidney dysfunction; (iii) with acute or chronic infectious diseases; (iv) with surgical operation in the past 6 months; (v) pregnant women or lactating mothers. Additionally, a total of 50 healthy people were screened as healthy controls (HCs), whose age and sex were matched with AMI patients. The eligible criteria for HCs were: (i) without abnormalities in recent physics examination; (ii) ≥18 years old; (iii) willing to cooperate with this study. The exclusion criteria for AMI patients were suitable for the HCs as well. The Ethics committee of the Central Hospital of Wuhan, Tongji Medical College, Huazhong University of Science and Technology gave approval to this study. The informed consent was obtained from all AMI patients or their families, and HCs.

### 2.2 Data documentation

Characteristics of AMI patients were collected for analyses, which included age, sex, body mass index (BMI), marital status, education level, location, smoke, hypertension, hyperlipidemia, diabetes, diagnosis, culprit lesion, multivessel disease, blood biochemical indexes, symptom-to-balloon time, thrombus aspiration, number of implanted stents, stent diameter, total stent length, and infarct size.

### 2.3 Sample collection and detection

Peripheral blood samples from AMI patients were collected at admission and after PCI (at the time of discharge); while samples from HCs were collected at the time of enrollment. After sample collection, the serum was immediately isolated. Then, the exosomes were separated from the serum through Total Exosome Isolation (from serum) (No. Cat. 4478360, Invitrogen™, Waltham, USA). All test procedures were performed in strict accordance with the instructions.

Serum exosomal miR-186-5p was detected using a reverse transcriptase-quantitative polymerase chain reaction (RT-qPCR) assay. The U6 was used as the internal reference, and the final results were calculated using the 2^−ΔΔCt^ method. The primer sequences of serum exosomal miR-186-5p and U6 were the same as in our previous study (17).

### 2.4 Follow-up and prognosis analyses

AMI patients underwent routine follow-ups until July 2023. Follow-up visits were conducted once per month in the first three months, and then every three months thereafter. During the follow-up, MACE was recorded, which contained recurrent thrombosis in stents, recurrent myocardial infarction, revascularization, malignant arrhythmia, cerebral infarction, and cardiac death (19). Besides, the accumulating MACE rates were calculated for prognosis analyses. In this study, the serum exosomal miR-186-5p at admission and after PCI of AMI patients was divided into high and low levels, utilizing the maximum value of 3.39 in HCs. The decrement percentage of serum exosomal miR-186-5p was divided into <median (35%) decrement and ≥median (35%) decrement.

### 2.5 Statistical analyses

SPSS version 26.0 (IBM, USA) was used for the data process. The Wilcoxon rank sum test was used to compare serum exosomal miR-186-5p between AMI patients and HCs, as well as compare serum exosomal miR-186-5p between AMI patients with different characteristics (except for education level using Spearman test and culprit lesion using Kruskal–Wallis *H*-test). The receiver operating characteristic (ROC) curve was used to show the distinguishing ability of serum exosomal miR-186-5p to separate HCs from AMI patients. The Spearman test was performed to analyze the correlation of serum exosomal miR-186-5p with continuous variables in AMI patients. The Wilcoxon signed-rank test was used for comparing serum exosomal miR-186-5p at admission and after PCI. The Kaplan-Meier curves were displayed to show the accumulating MACE rates. The Log-rank test was used to compare the accumulating MACE rates between AMI patients with different levels of serum exosomal miR-186-5p. A *P*-value less than 0.05 indicated statistical significance.

## 3 Results

### 3.1 Clinical information on AMI patients receiving PCI

The enrolled patients had a mean age of 61.4 ± 10.0 years, and there were 107 (71.3%) male patients. Meanwhile, 111 (74.0%) patients were diagnosed with STEMI, and 39 (26.0%) patients were diagnosed with NSTEMI. A total of 65 (43.3%) patients had multivessel disease. The median [interquartile range (IQR)] symptom-to-balloon time was 4.1 (2.5–7.9) hours. Additionally, the number of implanted stents was 1 in 117 (78.0%) patients, and it was 2 in 33 (22.0%) patients. Furthermore, the mean infarct size was 21.7 ± 8.1%. The mean left ventricular ejection fraction value was 43.5 ± 12.5%. The specific clinical features of AMI patients receiving PCI are listed in Table 1.

**Table 1:** Characteristics of AMI patients.

Characteristics	AMI patients (*N* = 150)
Age (year)	61.4 ± 10.0
Male	107 (71.3)
BMI (kg/m^2^)	25.5 (22.3–27.8)
Married	114 (76.0)
Education level	
Primary school	27 (18.0)
Middle school	46 (30.7)
High school	49 (32.7)
Undergraduate or above	28 (18.7)
Location	
Urban	134 (89.3)
Rural	16 (10.7)
Smoker	65 (43.3)
Hypertension	103 (68.7)
Hyperlipidemia	59 (39.3)
Diabetes	34 (22.7)
Diagnoses	
STEMI	111 (74.0)
NSTEMI	39 (26.0)
Culprit lesion	
LAD	61 (40.7)
LCX	38 (25.3)
RCA	51 (34.0)
Multivessel disease	65 (43.3)
Blood biochemical indexes	
Scr (μmol/L)	73.3 (64.3–89.7)
TG (mmol/L)	1.6 (0.9–2.3)
TC (mmol/L)	4.7 (3.8–5.4)
LDL-C (mmol/L)	3.2 (2.4–3.9)
HDL-C (mmol/L)	1.0 (0.9–1.2)
CRP (mg/L)	7.0 (4.6–9.9)
cTnI (ng/ml)	4.0 (2.6–5.5)
CK-MB (ng/ml)	28.1 (17.3–43.5)
eGFR (ml/min/1.73 m^2^)	90.0 (72.4–99.0)
Symptom-to-balloon time (h)	4.1 (2.5–7.9)
Thrombus aspiration	36 (24.0)
Number of implanted stents	
1	117 (78.0)
2	33 (22.0)
Stent diameter (mm)	3.0 (3.0–3.5)
Total stent length (mm)	33.0 (23.0–38.0)
Infarct size (%)	21.7 ± 8.1
Left ventricular ejection fraction (%)	43.5 ± 12.5

Kolmogorov–Smirnov test was used to normality test. Values were given as mean ± standard deviation for normally distributed continuous variables, median (interquartile range) for non-normally distributed continuous variables, or numbers (percentage) for categorical variables. AMI, acute myocardial infarction; BMI, body mass index; STEMI, ST-segment elevation myocardial infarction; NSTEMI, non-ST-segment elevation myocardial infarction; LAD, left anterior descending artery; LCX, left circumflex artery; RCA, right coronary artery; Scr, serum creatinine; TG, triglycerides; TC, total cholesterol; LDL-C, low-density lipoprotein cholesterol; HDL-C, high-density lipoprotein cholesterol; CRP, C-reactive protein; cTnI, cardiac troponin I; CK-MB, creatine kinase-MB; eGFR, estimated glomerular filtration rate.

### 3.2 Comparison of serum exosomal miR-186-5p between AMI patients receiving PCI and HCs

**Figure 1:**
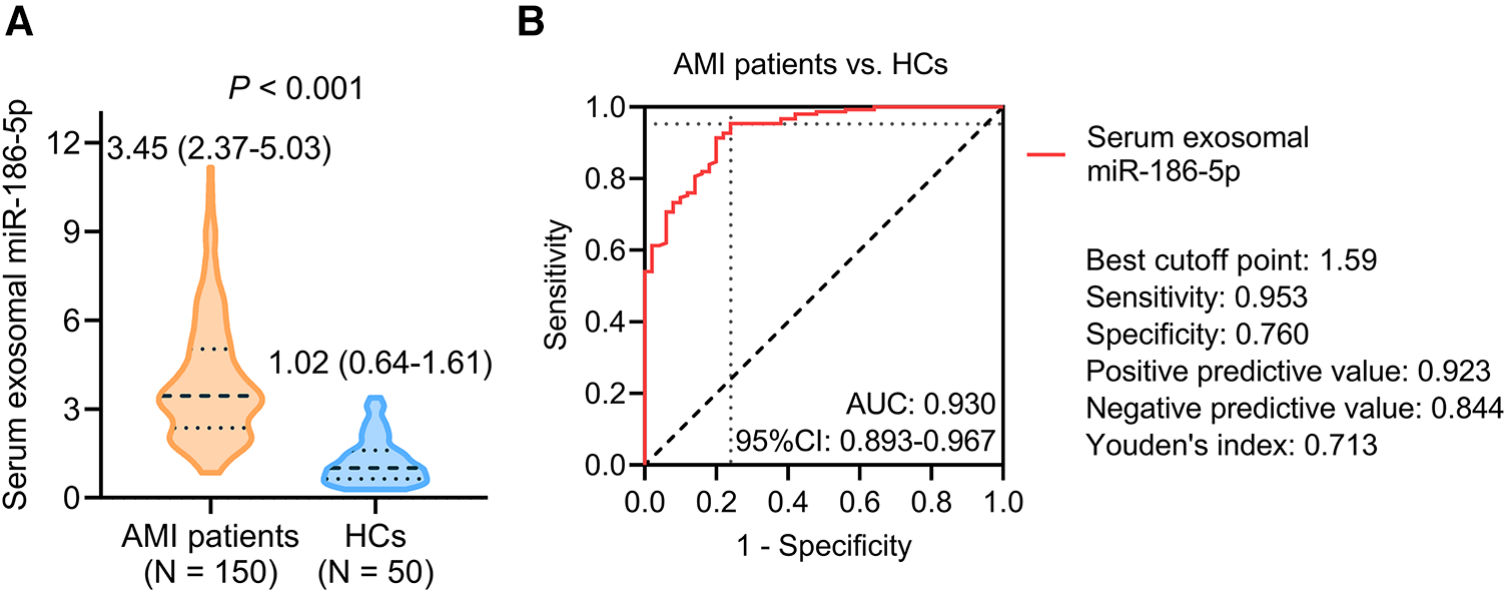
Serum exosomal miR-186-5p was higher in AMI patients receiving PCI than HCs. Comparison (**A**) and discriminative ability (**B**) of serum exosomal miR-186-5p between AMI patients receiving PCI and HCs.

Serum exosomal miR-186-5p was raised in AMI patients receiving PCI versus (vs.) HCs [median (IQR): 3.45 (2.37–5.03) vs. 1.02 (0.64–1.61)] (*P *< 0.001) (Figure 1A). According to the ROC curve, serum exosomal miR-186-5p had an acceptable ability to distinguish AMI patients receiving PCI from HCs, with an area under the curve (AUC) [95% confidence interval (CI)] of 0.930 (0.893–0.967). The value of serum exosomal miR-186-5p at the best cutoff point was 1.59 (sensitivity: 0.953, specificity: 0.760) (Figure 1B).

### 3.3 Relationship of serum exosomal miR-186-5p with clinical properties in AMI patients receiving PCI

In terms of continuous variables, serum exosomal miR-186-5p was positively linked with BMI (*r *= 0.162, *P *= 0.048), serum creatinine (Scr) (*r *= 0.189, *P *= 0.021), total cholesterol (TC) (*r *= 0.179, *P *= 0.029), and C-reactive protein (CRP) (*r *= 0.193, *P *= 0.018) in AMI patients receiving PCI. In contrast, serum exosomal miR-186-5p was inversely linked with the estimated glomerular filtration rate (eGFR) (*r *= −0.185, *P *= 0.023). However, serum exosomal miR-186-5p was not associated with other continuous variables, including age, triglycerides, low-density lipoprotein cholesterol, high-density lipoprotein cholesterol, cardiac troponin I, creatine kinase-MB, symptom-to-balloon time, stent diameter, total stent length, and infarct size (all *P *> 0.05) (Table 2).

**Table 2:** Correlation of serum exosomal miR-186-5p with continuous variables in AMI patients.

Variables	*r*	*P*-value
Age (year)	0.106	0.195
BMI (kg/m^2^)	0.162	0.048
Scr (μmol/L)	0.189	0.021
TG (mmol/L)	0.152	0.063
TC (mmol/L)	0.179	0.029
LDL-C (mmol/L)	0.158	0.053
HDL-C (mmol/L)	−0.055	0.502
CRP (mg/L)	0.193	0.018
cTnI (ng/ml)	0.132	0.107
CK-MB (ng/ml)	0.110	0.181
eGFR (ml/min/1.73 m^2^)	−0.185	0.023
Symptom-to-balloon time (h)	−0.066	0.420
Stent diameter (mm)	−0.025	0.763
Total stent length (mm)	0.130	0.112
Infarct size (%)	0.133	0.106

AMI, acute myocardial infarction; BMI, body mass index; Scr, serum creatinine; TG, triglycerides; TC, total cholesterol; LDL-C, low-density lipoprotein cholesterol; HDL-C, high-density lipoprotein cholesterol; CRP, C-reactive protein; cTnI, cardiac troponin I; CK-MB, creatine kinase-MB; eGFR, estimated glomerular filtration rate.

Regarding categorical variables, serum exosomal miR-186-5p was only linked with the diagnosis of STEMI (*P *= 0.034). But it was not linked to sex, marital status, educational level, location, smoking status, hypertension, hyperlipidemia, diabetes, diagnoses, culprit lesion, multivessel disease, thrombus aspiration, or number of implanted stents in AMI patients receiving PCI (all *P *> 0.05) (Table 3).

**Table 3:** Comparison of serum exosomal miR-186-5p in AMI patients with different categorical variables.

Variables	Serum exosomal miR-186-5p	*P*-value
Sex		0.964
Female	3.41 (2.19–5.21)	
Male	3.48 (2.37–4.99)	
Married		0.424
No	3.50 (2.42–5.74)	
Yes	3.43 (2.32–4.85)	
Education level		0.422
Primary school	3.48 (3.00–6.02)	
Middle school	3.41 (2.06–4.63)	
High school	3.48 (2.93–5.43)	
Undergraduate or above	3.13 (1.82–4.57)	
Location		0.148
Urban	3.42 (2.34–4.85)	
Rural	4.76 (2.53–6.97)	
Smoker		0.238
No	3.35 (2.20–5.13)	
Yes	3.82 (2.90–5.01)	
Hypertension		0.213
No	3.30 (2.09–4.97)	
Yes	3.51 (2.67–5.04)	
Hyperlipidemia		0.098
No	3.41 (2.18–4.57)	
Yes	3.66 (2.86–5.97)	
Diabetes		0.145
No	3.41 (2.18–5.04)	
Yes	3.79 (3.16–4.76)	
Diagnoses		0.034
STEMI	3.48 (2.67–5.33)	
NSTEMI	3.22 (1.80–4.26)	
Culprit lesion		0.250
LAD	3.48 (2.46–5.24)	
LCX	3.63 (2.87–5.74)	
RCA	3.04 (2.11–4.79)	
Multivessel disease		0.105
No	3.35 (1.97–5.26)	
Yes	3.66 (2.96–4.64)	
Thrombus aspiration		0.740
No	3.48 (2.20–5.00)	
Yes	3.37 (2.91–5.21)	
Number of implanted stents		0.176
1	3.35 (2.16–5.23)	
2	3.73 (3.06–4.57)	

Values were given as median (interquartile range). AMI, acute myocardial infarction; STEMI, ST-segment elevation myocardial infarction; NSTEMI, non-ST-segment elevation myocardial infarction; LAD, left anterior descending artery; LCX, left circumflex artery; RCA, right coronary artery.

### 3.4 Longitudinal change of serum exosomal miR-186-5p in AMI patients receiving PCI

**Figure 2:**
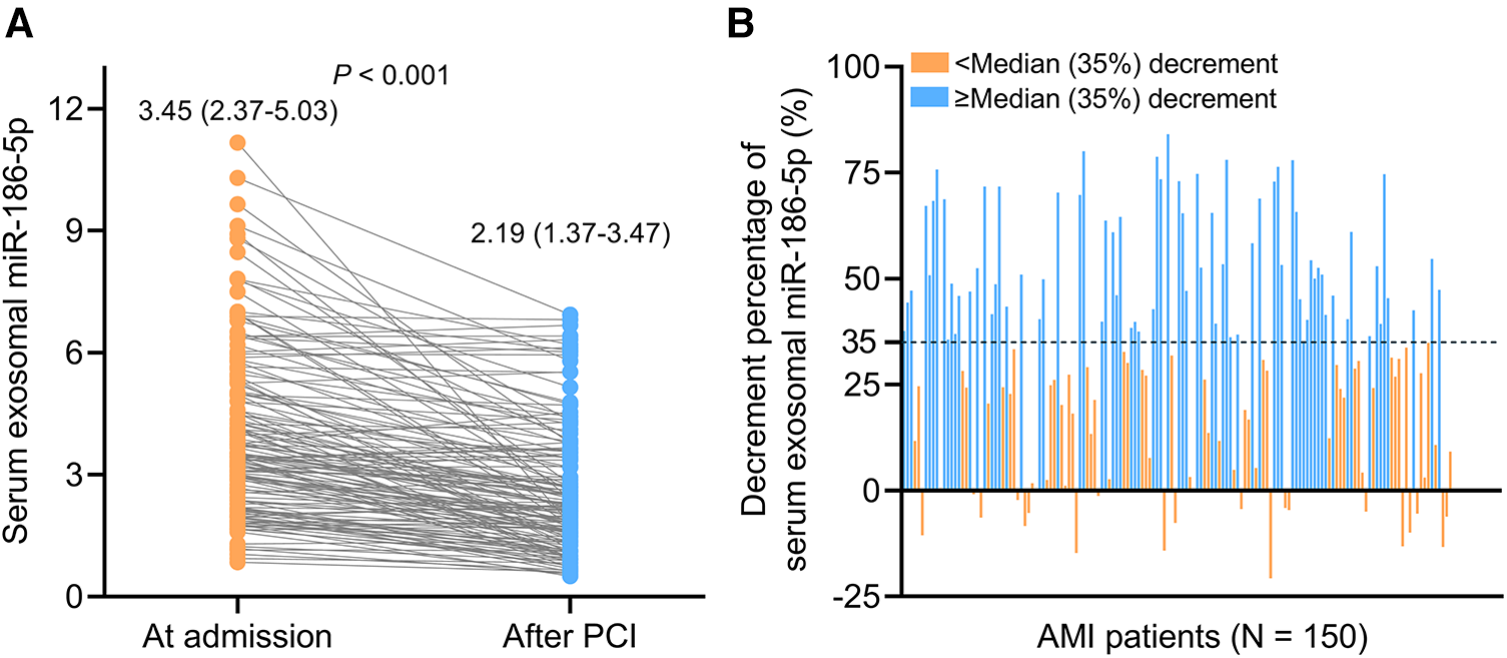
Serum exosomal miR-186-5p was decreased after PCI in AMI patients. Longitudinal change of serum exosomal miR-186-5p after PCI (**A**); exhibition of AMI patients receiving PCI with the decrement percentage of serum exosomal miR-186-5p < or ≥ median (35%) decrement (**B**).

Serum exosomal miR-186-5p was decreased after PCI [median (IQR): 2.19 (1.37–3.47)] compared to its level at admission [median (IQR): 3.45 (2.37–5.03)] in AMI patients (*P *< 0.001) (Figure 2A). The decrement percentage of serum exosomal miR-186-5p after PCI was divided into <median (35%) and ≥median (35%) decrement, which was exhibited in Figure 2B.

### 3.5 Correlation of serum exosomal miR-186-5p with MACE in AMI patients receiving PCI

**Figure 3:**
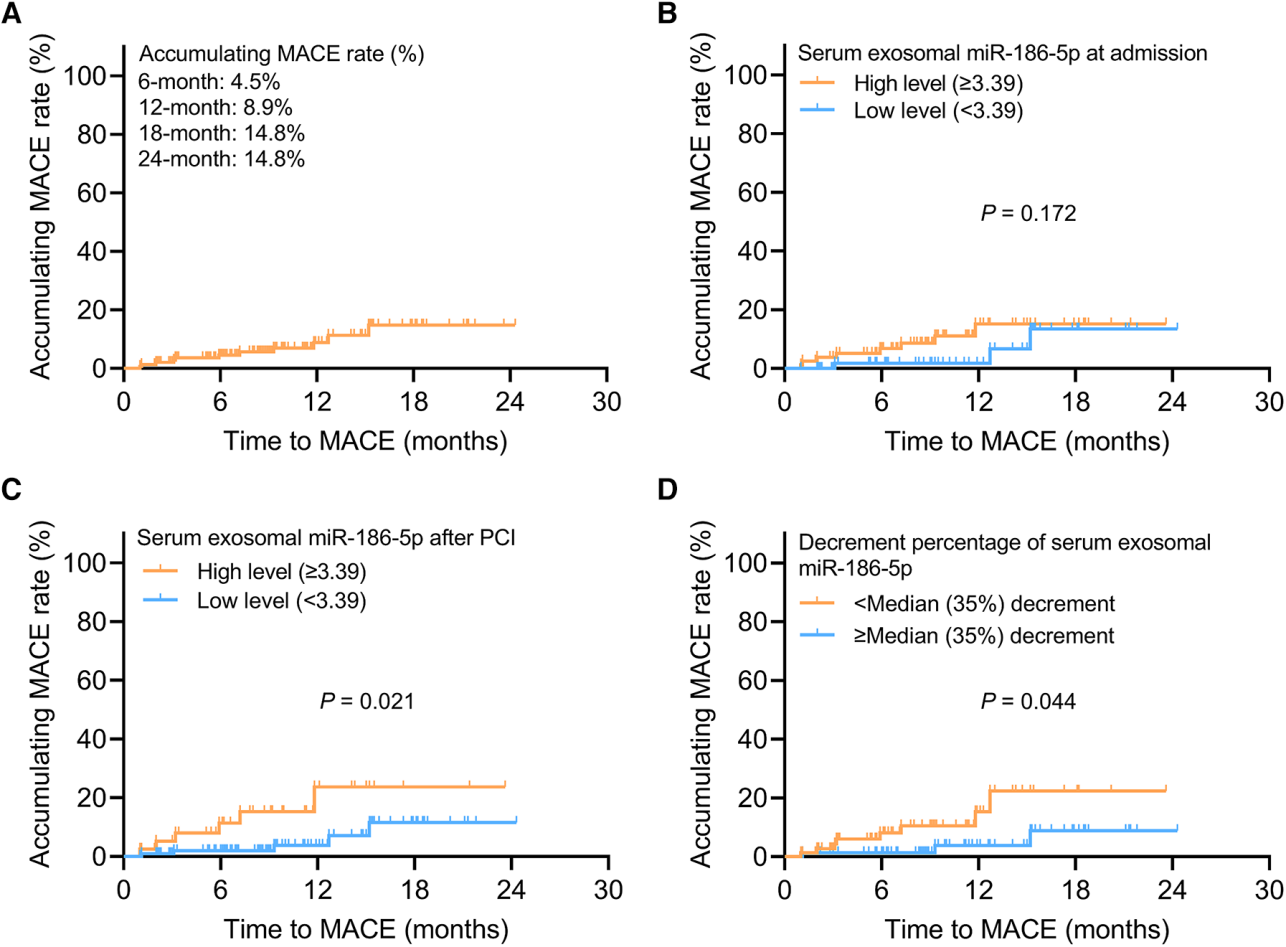
High level serum exosomal miR-186-5p or decrement percentage <median (35%) decrement after PCI estimated increased MACE in AMI patients. Exhibition of accumulating MACE rate (**A**); relationship of high level (≥3.39) serum exosomal miR-186-5p at admission (**B**) and after PCI (**C**) with MACE; relationship of serum exosomal miR-186-5p decrement percentage <median (35%) decrement with MACE (**D**) in AMI patients receiving PCI.

The 6-, 12-, 18-, and 24-month accumulating MACE rates were 4.5%, 8.9%, 14.8%, and 14.8%, respectively, in AMI patients receiving PCI (Figure 3A). Serum exosomal miR-186-5p at admission was not related to MACE (*P *= 0.172) (Figure 3B). Notably, high level (≥3.39) serum exosomal miR-186-5p after PCI was related to increased MACE in AMI patients (*P *= 0.021) (Figure 3C). Decrement percentage of serum exosomal miR-186-5p <median (35%) decrement was correlated with elevated MACE in AMI patients receiving PCI (*P *= 0.044) (Figure 3D).

## 4 Discussion

The dysregulation of miR-186-5p has been discovered in various cardiovascular diseases, according to previous studies (20–22). However, miR-186-5p is detected in serum in these previous studies (20–22). It should be mentioned that in our recently published study, we find out that exosomal miR-186-5p promotes atherosclerosis by enhancing VSMC viability and invasion, indicating an engagement of exosomes-delivered miR-186-5p in cardiovascular diseases (17). On the other hand, due to the protection of the lipid bilayer of exosomes, serum exosomal miRNAs are steady compared to free serum miRNAs (23). Hence, serum exosomal miR-186-5p may be a more credible marker in patients with cardiovascular diseases (9). In this research, it was discovered that serum exosomal miR-186-5p was increased in AMI patients receiving PCI vs. HCs. A potential explanation might be that exosomal miR-186-5p might modulate the LOX-1 and SHIP2-mediated PI3K/AKT/mTOR pathways to facilitate lipid accumulation and promote viability and invasion in VSMCs, thereby contributing to atherosclerosis, which led to the occurrence of AMI (15, 17). Therefore, serum exosomal miR-186-5p reflected an increased risk of AMI.

The current study further explored the relationship of serum exosomal miR-186-5p with clinical properties in AMI patients receiving PCI. Firstly, it was discovered that serum exosomal miR-186-5p was positively related to BMI and TC in AMI patients receiving PCI. A potential reason might be that exosomal miR-186-5p might facilitate lipid accumulation, which further led to a higher BMI and TC (15). Secondly, serum exosomal miR-186-5p was positively correlated with Scr and inversely linked with eGFR in AMI patients receiving PCI. It would be due to the fact that exosomal miR-186-5p might modulate Smad5 and toll-like receptor 7/8 axis to induce renal inflammation and fibrosis, which impaired renal function and resulted in a higher Scr and lower eGFR (24, 25). Thirdly, serum exosomal miR-186-5p was positively related to CRP in AMI patients receiving PCI. A reason behind this could be that exosomal miR-186-5p might regulate the PTEN/AKT pathway to facilitate the secretion of proinflammatory cytokines, which aggravated the inflammation, thereby leading to higher CRP (26). Fourthly, serum exosomal miR-186-5p was related to the diagnosis of STEMI in AMI patients receiving PCI. It was speculated that exosomal miR-186-5p could facilitate atherosclerosis to exacerbate the occlusion of blood vessels, leading to STEMI (17).

The change of miR-186-5p after PCI in patients with cardiovascular diseases has been reported by one previous study, which discloses that serum miR-186-5p declined after PCI in ACS patients (22). This previous study also indicates that higher serum miR-186-5p at admission estimates a higher MACE risk in ACS patients after PCI (22). In our study, we discovered that serum exosomal miR-186-5p was reduced after PCI in AMI patients. A probable explanation might be that exosomal miR-186-5p could facilitate the progression of AMI, while disease progression was attenuated after PCI, which contributed to a lower exosomal miR-186-5p (17). Apart from this finding, we also discovered that high level serum exosomal miR-186-5p after PCI and its decrement percentage <median (35%) decrement was related to increased MACE in AMI patients. The potential arguments would be that: (1) exosomal miR-186-5p might regulate C1q/TNF-related protein 3 and Yin Yang 1 pathway to aggravate myocardial ischemia/reperfusion-induced injury after PCI, which led to the occurrence of MACE (27, 28). (2) as mentioned above, exosomal miR-186-5p could regulate various pathways to facilitate atherosclerosis to induce MACE (15, 17). Therefore, exosomal miR-186-5p estimated increased MACE in AMI patients receiving PCI.

It should be clarified that immune cells play a fundamental role in the pathology and progression of AMI (29). Additionally, miR-186-5p shows the potential to regulate the inflammatory response, migration, proliferation, foaming, and polarization of immune cells (15, 27, 30, 31). Therefore, we speculated that miR-186-5p might also regulate the functionality of immune cells to engage in AMI pathology, which could be study direction for our subsequent studies. Moreover, in our previous study, we discover that macrophage-derived exosomal miR-186-5p is involved in the pathology and progression of AMI (17). In addition, macrophages play a fundamental role in regulating inflammation and atherogenesis, and one previous study indicates that miR-186-5p regulates LOX-1 to enhance macrophage foaming, thereby accelerating atherosclerosis (15, 32). Therefore, it might be meaningful to explore the abundance of macrophages in AMI patients, and the correlation between exosomal miR-186-5p and the abundance of macrophages could also be explored by subsequent studies. Apart from macrophages, regulatory T cells (Tregs) regulates cardiac fibrosis, inflammation, and angiogenesis to engage in AMI (33). Hence, further studies should also explore the abundance of Tregs in AMI patients.

Several limitations should be mentioned in the current study. (1) this was a single-center study; thus, selection bias would unavoidably exist. (2) the follow-up duration could be prolonged to explore the long-term prognostic implication of serum exosomal miR-186-5p in AMI patients receiving PCI. (3) the number of HCs was small and unmatched to the number of AMI patients receiving PCI, which might affect the results. (4) immune cells played fundamental role in the pathology and progression of AMI; further studies should explore the longitudinal changes of distinct immune cells in AMI patients, and the effect of exosomal miR-186-5p on the functionality of distinct immune cells should be further investigated as well.

In conclusion, serum exosomal miR-186-5p is reduced after PCI; its post-PCI high level and minor reduction predicts increased MACE risk in AMI patients.

## Data Availability

The original contributions presented in the study are included in the article/Supplementary Material, further inquiries can be directed to the corresponding authors.
